# Ethics and responsibility in biohybrid robotics research

**DOI:** 10.1073/pnas.2310458121

**Published:** 2024-07-23

**Authors:** Rafael Mestre, Aníbal M. Astobiza, Victoria A. Webster-Wood, Matt Ryan, M. Taher A. Saif

**Affiliations:** ^a^Agents, Interaction and Complexity Group, School of Electronics and Computer Science, University of Southampton, Southampton SO17 1BJ, United Kingdom; ^b^Politics and International Relations Department, University of Southampton, Southampton SO17 1BJ, United Kingdom; ^c^Centre for Democratic Futures, University of Southampton, Southampton SO17 1BJ, United Kingdom; ^d^Centre for Robotics, University of Southampton, Southampton SO17 1BJ, United Kingdom; ^e^Institute for Life Sciences, University of Southampton, Southampton SO17 1BJ, United Kingdom; ^f^Department of Public Law, University of the Basque Country/Euskal Herriko Unibertsitatea, Donostia 20018, Spain; ^g^Department of Mechanical Engineering, Carnegie Mellon University, Pittsburgh 15213, Pennsylvania; ^h^Department of Biomedical Engineering, Carnegie Mellon University, Pittsburgh 15213, Pennsylvania; ^i^The Robotics Institute, Carnegie Mellon University, Pittsburgh 15213, Pennsylvania; ^j^The McGowan Institute for Regenerative Medicine, University of Pittsburgh, Pittsburgh 15213, Pennsylvania; ^k^Democratic Innovations Research Unit, Goethe-Universität, Frankfurt am Main 60323, Germany; ^l^Mechanical Science and Engineering, University of Illinois at Urbana-Champaign, Urbana 61801, Illinois

**Keywords:** biohybrid robots, ethics, policy, governance, synthetic biology

## Abstract

The industrial revolution of the 19th century marked the onset of an era of machines and robots that transformed societies. Since the beginning of the 21st century, a new generation of robots envisions similar societal transformation. These robots are biohybrid: part living and part engineered. They may self-assemble and emerge from complex interactions between living cells. While this new era of living robots presents unprecedented opportunities for positive societal impact, it also poses a host of ethical challenges. A systematic, nuanced examination of these ethical issues is of paramount importance to guide the evolution of this nascent field. Multidisciplinary fields face the challenge that inertia around collective action to address ethical boundaries may result in unexpected consequences for researchers and societies alike. In this Perspective, we i) clarify the ethical challenges associated with biohybrid robotics, ii) discuss the need for and elements of a potential governance framework tailored to this technology; and iii) propose tangible steps toward ethical compliance and policy formation in the field of biohybrid robotics.

Over recent decades, the world of robotics has seen tremendous advancements, impacting numerous facets of our lives. A promising frontier in this field is biohybrid robotics, a concept that merges biological tissue or cells with artificial components. This hybridization not only has the potential to redefine our understanding of robotics but also presents novel ethical issues due to the intrinsic complexity of combining living and nonliving entities. While the ethical dilemmas associated with biohybrid robotics resonate with challenges seen in fields like biomedicine, conventional robotics, or artificial intelligence, the unique amalgamation of living and nonliving components in biohybrid robots, also called biorobots, breeds its own set of ethical complexities that warrant a tailored investigation. Delaying consideration of these ethical issues could lead to public misconceptions, potentially undermining the public’s trust and investment in research and development in this area.

Consider a speculative, yet plausible, scenario involving biohybrid robotics. You are the chief researcher at SkyBioTech, a leading biohybrid robotic company. You are charged with creating a unique breed of biohybrid drones incorporating muscle tissues from a certain bird species renowned for their exceptional flight endurance. You and your team are working to transform the aerospace sector with these drones, capable of extensive, energy-efficient flight times, which could revolutionize various sectors from environmental surveillance to search and rescue operations. However, unlike traditional robotic designs, your work involves sourcing embryonic cells from these migratory birds, adding a unique bioethical dimension to your technological development. Your team has ensured that this procedure is done without harm in a nonintrusive way, but external voices have raised complaints about the long-term risks to these birds. This species had been relatively undisturbed by humans but, because of this new application, ecologists are afraid that it could lead to furtive hunting and endangerment.

At first glance, this situation presents an ethical challenge familiar to many technologies: balancing benefits to humanity against potential harm to the environment. Yet, the blend of biological and mechanical components in an autonomous biohybrid robot adds further layers to this ethical predicament, which might remain unattended at a first glance. These novel entities challenge our traditional distinctions between life and machine and demand a nuanced ethical evaluation that respects both the autonomy and integrity of the biological component. For instance, what should our relationship to this form of biohybrid entity be? How does one make a balanced and equitable measurement of the value of a biohybrid robot’s life-like attributes against its purely mechanical properties? The former raises issues around biological integrity, animal rights, and potentially the sanctity of life itself, making the moral calculus far more complex than for conventional robots. As we blend life and machine, how does this blur the lines of agency, autonomy, and the sanctity of life, particularly when these creations take on life-like attributes?

Building upon the earlier speculative scenario at SkyBioTech, the complexity of ethical and ecological considerations can become further pronounced. For instance, experts might estimate upon deployment of these biohybrid birds for different applications that, for every ten drones deployed, one bird of a different species is affected due to the disruption of their ecosystem. You find yourself asking: Is it morally justified to facilitate life-saving search and rescue operations while potentially hurting another species? During the research and development phase, you may have accepted the uncomfortable reality of harming one bird for every ten drones. However, as the demand for and scale of operation of your drones increase, leading to the production of more drones, the number of affected birds could rise, even if significant improvements in technology and ethical considerations have greatly improved this ratio. This exponential growth might even risk causing a shift in the species’ population dynamics and ecosystem balance. How does your organization navigate this ethical tightrope? When should the benefits to humanity outweigh the potential harm to another species? You are the first one to come across this type of conundrum in a real application, as nobody foresaw the unique ethical dilemmas of this biological integration. How will the decisions that we make now toward these biological entities shape our future relationship between humans, the natural world, and potential biological robots?

These questions, while echoing ethical debates in domains such as AI ethics (concerning consciousness and autonomy), biomedical ethics (dealing with the sanctity of animal and human life) or classical robotics (dignity and rights of human-like robots), also extend beyond these frameworks due to the unique intersection of life and machine in biohybrid robots. The intertwining of biological and mechanical parts introduces unique ethical dimensions that other technologies do not engage within the same way, making the development of ethics specific to biohybrid robotics indispensable. In biohybrid robots, questions arise about the integrity of life, the status and rights of semiliving beings, and the potential long-term impacts on natural ecosystems. Considerations must also be given to the “unknown unknowns,” i.e., the impacts and ethical dilemmas that we cannot predict due to the novel integration of life and machine, and the potential for these systems to evolve in unforeseen ways.

Exploring such dilemmas, even if only as thought experiments, provides valuable insights into the reasoning and decision-making processes specific to biohybrid robotics. Such thought experiments encourage us to consider the consequences of our choices and evaluate scenarios where established rules or principles might apply (1). In reading the previous thought experiment, we came up with a series of ethical questions that emerged naturally from the scenario. Without realizing, we have taken the standpoints of (at least) four different schools of thought in ethics that have added more layers to the tension of different ethical interpretations. These four schools of thoughts are consequentialism (2), deontologism (3, 4), virtue ethics (5) and ethics of responsibility (6). These schools provide varied yet valuable lenses through which we can examine the ethics of biohybrid robotics.

The development of disruptive technologies, such as biohybrid robotics, will present researchers with dilemmas like the one above. These questions underline the need for an ethical framework that acknowledges the novel complexities of biohybrid robotics. There is, therefore, a pressing need for interdisciplinary dialog. This dialog must involve ethicists, scientists, engineers, legal experts, and the wider public to formulate robust ethical guidelines and regulatory frameworks. These guidelines should reflect the complexity of biohybrid systems and anticipate the broad range of potential implications their use may entail, from environmental impacts to societal perceptions and acceptance. In this paper, we explore responsible research and innovation in the context of biohybrid robotics, with a specific focus on the nuanced interplay of consequentialism, deontologism, virtue ethics, and ethics of responsibility, and we seek to contribute to that necessary dialog by discussing the ethical implications and considerations surrounding biohybrid robots. As we embark on this journey of exploration, we invite you to consider the fundamental question: What do we build robots for? And why should we build them?

## 1. Biohybrid Robotics: Living Robots and Living Machines

Biohybrid robotics is an emerging field at the intersection of robotics and bioengineering that combines living materials and organisms with synthetic robotic components. Through the use of living materials as the structure, sensor, actuator, or controller of a robotic system, researchers are able to harness several advantages, including self-healing, adaptability, natural compliance, and sensor resolution, with the robustness of synthetic systems (7–10). Living materials in biohybrid robots, or biorobots, have included muscle cells as actuators, including those from primary, cell line, or induced pluripotent stem cell sources, neurons as motor controllers, sensory cells either in a dish or through organ isolation, bacteria, spermatozoa, protozoa, and larger intact living organisms (11).

The past several decades have seen numerous advances in the field of biohybrid robotics. For example, sensors based on recording electrical signals from sensory cells or isolated insect antennae have been demonstrated to improve chemical sensing in complex environments (12–14). Furthermore, biohybrid robots or actuators that harness living muscle as small-scale compliant actuators have been developed that are capable of crawling (15–21), swimming (22–29), gripping (27, 30), pumping (25, 31–34), and sensing (12–14, 35–40). Living neurons can even be used to control mobile robot platforms (41, 42). Additional performance capabilities can be achieved by combining multiple living components within the biohybrid device, such as through the coculture or intact isolation of neuromuscular systems for motor control and robot actuation (20, 21, 42). Although a complete review of the field is beyond the scope of this work, interested readers are directed to several recent reviews on the state of biohybrid robotics (7–10) and future challenges facing the field (11).

This field is advancing rapidly with many contributing labs around the world, however, there are still major challenges to overcome. The most daunting one is the lack of any quantitative predictive “law” of living cells and their interactions, unlike the laws of interaction between two charges. Many biohybrid robots have engineered scaffolds and extracellular matrix (ECM). This involves another level of interaction between cells, ECM, and their scaffolds. The field thus far has relied on empiricism and intuition to design and develop various biohybrid robots, a few of which are mentioned above. The lack of predictive laws for biohybrid robots originates from a limited understanding of living cells themselves due to their unprecedented richness and complexities. This implies that two biohybrid robots, manufactured by identical processes, are not guaranteed to be even nominally identical, unlike two engineering products of the same model and maker. The field is thus faced with a new paradigm: Robots gain living ingredients empowered through millions of years of evolution, but engineering precision is lost in the exchange.

Another important aspect of the field of biohybrid robotics distinguishes it from solid-state robots. Biohybrid robots serve as a platform for understanding life itself. Developing components for these robots, such as muscle actuators, neuronal circuits as sensors, or neuro-muscular junctions to form intelligent robots, requires a deeper exploration of cells and their behavior beyond traditional Petri dish platforms. Each biohybrid robot emerges into a “being” mimicking “development in vitro.” Thus, irrespective of its function as a robot, its journey through this development delivers a wealth of new knowledge. Recent publication rates provide evidence of the popularity of biohybrid robots among the scientific community worldwide.

**Fig. 1.**
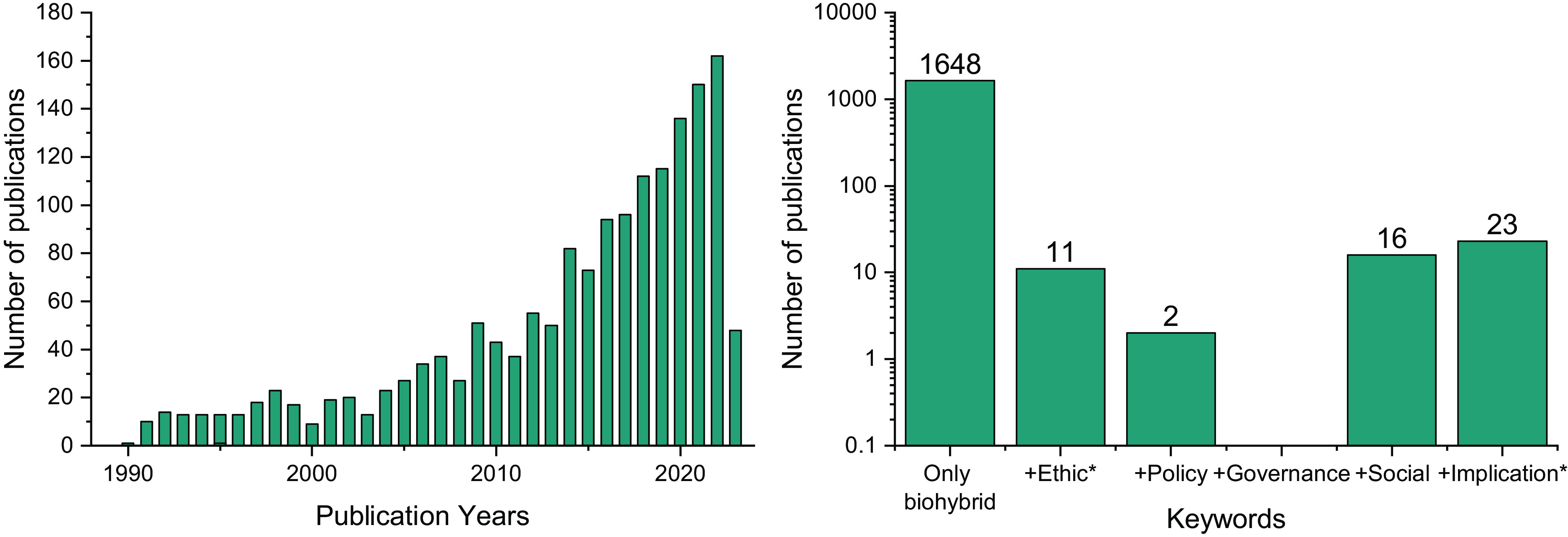
(*Left*) Number of publications over the years related to biohybrid robotics, according to our set of keywords. (*Right*) Total number of publications on the topic of biohybrid devices (1648) that also contain in the abstract, title, or keywords the words: ethic*, policy, governance, social, or implication*. Both searches were performed on May 17, 2023.

The publication of research related to biohybrid robots has increased continuously over the last decade. A search in the Web of Science engine^*^ returns the number of publications displayed in Fig. 1, where we can see that the number of papers in this discipline has significantly increased in the last decade, soon to reach an exponential rate. Despite this increase, the analysis of ethical implications has barely been touched upon. Out of the 1,648 publications that contained the set of keywords we used for our search, only 11 also contained the keyword ethic* in their abstract or title. Although this does not mean that only 11 publications considered ethical implications, those are the only papers that considered ethical analysis so central to their work to be included in their abstract. After filtering out false positives, only 5 are relevant to this field^†^ and dedicate several sentences or paragraphs to ethical considerations of this type of research. We briefly review them here.^‡^

Raman and Bashir and Kamm and Bashir provide an excellent review of the field of biohybrid systems or living machines (43, 44). Although they dedicate a subsection to ethical implications, this is not the central contribution of their work and does not contain contributions from experts in bioethics and policy making. In similar publications, however, the authors make a positive call for ethico-technical evaluation of the discipline, arguing that “[we] may benefit from an intentional structure for identifying underlying, competing values and ethical principles” and that “[t]he development of such a framework and an ethics code of conduct […] is an imperative task” (45). From the other extreme, in philosophy, Gómez-Márquez has published an article in which he proposed a new domain, Lithbea, to refer to synthetic/artificial life forms like xenobots or biobots (46).

Other works like those by Levin, Bongard and Lunshof and Coughlan and Leins have addressed in more detail the ethical and social implications of these technologies, but focused on specific systems like the xenobots and the implications of computer-designed organisms (47, 48). As such, both are short, and a comprehensive treatment of the topic for the wide and growing audience of interest does not exist. Last, two works from Xu et al. require special mention. Although they were not collected in our initial literature search due to being in the form of a preprint and submitted versions, we acknowledge their relevance to our study (49, 50). There, the authors review the ethical implications, considerations, and implications of biohybrid jellyfish research and, more generally, of invertebrate research. Although focused on a subdiscipline of biohybrid robotics that uses a top–down approach (e.g., using animals directly to fabricate biorobots), many of their recommendations could be extrapolated to bottom–up research (e.g., using extracted or lab-grown tissue to fabricate biorobots), such as including ethics statements in research papers, cost–benefit analysis, and stricter scientific justification.

## 2. Ethical Issues Associated with Biohybrid Robotics

Technology, since *Homo Habilis*, has coevolved with us and has favored our adaptation and improved our well-being (51). As with any other technology, there is no reason to believe that biorobots, simply because they are a new technology, would not be beneficial to society. Despite these potential benefits, we need to understand and prioritize the ethical values concerning the usage of biorobots in order to mitigate possible risks. In response to this, we propose a governance structure that facilitates democratic regulation and oversight over this novel technology.

We categorize the ethical issues raised by biorobots or biohybrid robotics into the following themes: 1) Interactivity: interactions between biorobots, humans, and the environment; 2) integrability: assimilation of biorobots with human beings, for example, in vivo organs; and 3) ontological and moral status: the ethical implications if biorobots acquire moral status (for instance, through the development of consciousness^§^ in vitro). These themes can further lead to various ethical, social, and legal concerns, which become increasingly relevant when we consider that biorobots, as a technological instrument, can be applied across a broad spectrum of areas and for many purposes, some of which have yet to be conceived.

**Fig. 2.**
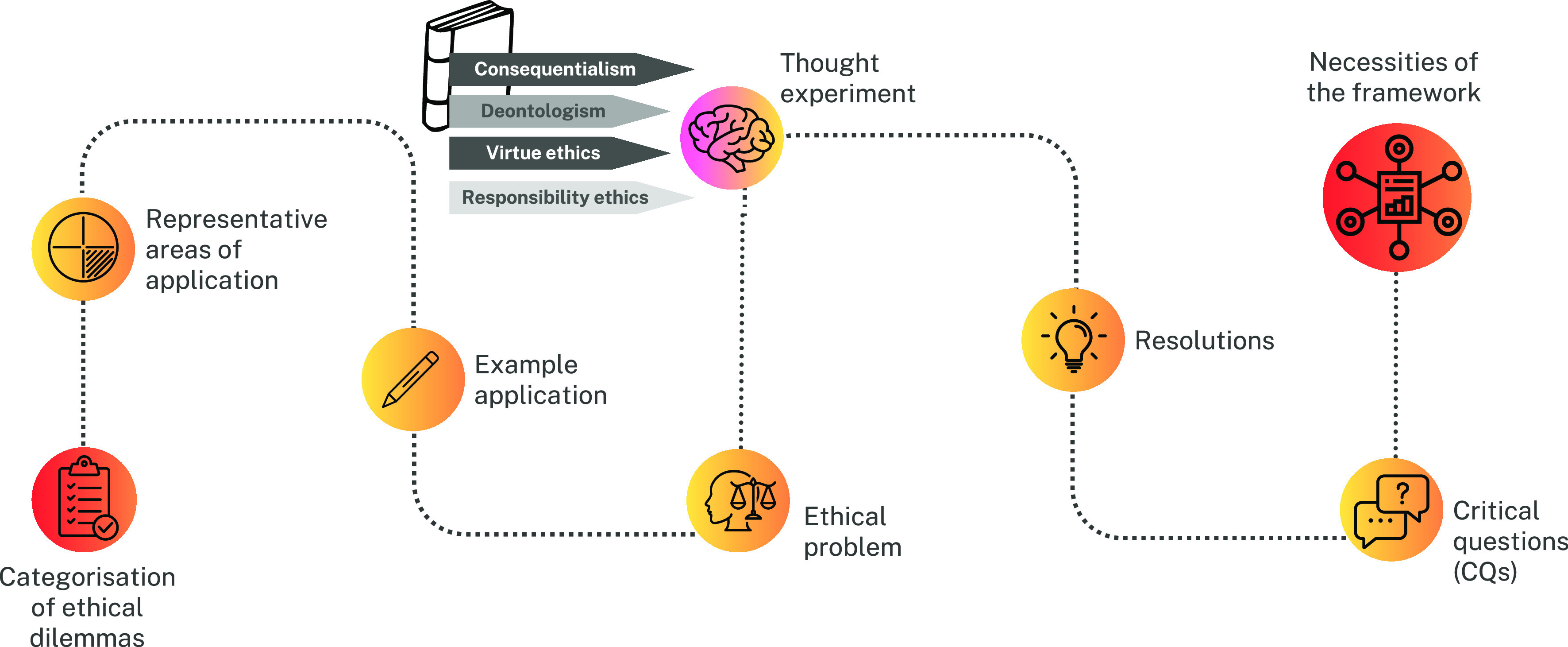
Scheme of our working process for the development of a framework for ethical and sociotechnical analysis of biohybrid robots.

As a groundwork for establishing a framework for the research, application, and governance of biohybrid robotics, we propose conducting several *gedankenexperimente*, or thought experiments. Using our categorization above, we select three cases of application of this technology that exemplify each of these ethical topics (Table 1). Subsequently, these examples are evaluated through the lens of four widely recognized normative ethical theories within moral philosophy. This enables us to formulate a list of critical questions (CQs) that those working with biorobots should contemplate. From this process, we derive a series of framework necessities (see scheme in Fig. 2).

**Table 1.** Three areas of application of biohybrid robots and examples

Categorization	Interactivity	Integrability	Moral status
Exemplary applications	E.g., waste removal, clean-up oceans	E.g., bioprostheses and hyperorgans	E.g., industrial or companionship robots that are half-biological (detection of consciousness and moral standing in biorobots)
Ethical problems	Release of biorobots without control, entering the trophic chain	Harm to humans in the long term, social inequalities, human enhancement	Achieving consciousness/moral status

### 2.1. Navigating Moral Dilemmas in Biohybrid Robotics Research.

In relation to each of these areas of application, it is interesting to summarize what four of the most prominent normative ethics theories^¶^ have to say in a table of trade-offs from different ethical perspectives. These theories include virtue ethics, deontology, consequentialism, and responsibility ethics (based on Hans Jonas’ ethics of responsibility).

Virtue ethics is traditionally associated with the following description: An action is right for someone only if a virtuous person would take that action. This theory holds that a virtuous person is someone with the habit of choosing a middle ground between two vices: excess and lack (*cf.* Aristotle, Aquinas, MacIntyre). We reach this point by exercising prudence (*phronesis*) and moderating our passions.

Deontological theories conclude that an action is right if and only if the action conforms to a right moral rule. For deontology (*cf.* Kant, Rawls), actions should be guided by moral principles that we would want to be universally applied. This is the categorical imperative, e.g., “I must not lie” because lying is not desirable as a universal rule of behavior.

Consequentialist theories spell out that an action is right if and only if the action produces the best available consequences. So, for this school (*cf.* Bentham, Mill), actions have no moral value in themselves. The value lies in their consequences: An act is morally good if its positive consequences are greater than its negative consequences. In one of its strands, consequentialism becomes utilitarianism when an action is evaluated depending on the pleasure/well-being it brings to most people.

Finally, in environmental ethics, the German philosopher Hans Jonas has focused on investigating the place of humanity in nature (6). Fundamental to Jonas’ work is the development of an ethics of responsibility in relation to nature and new technological breakthroughs. In diagnosing the situation of the biosphere and the situation of mankind and their welfare, Jonas suggests that the technology that has been created to satisfy human needs, and human value and function in modernity has taken on another character. In other words, modern technology is highly complex and can spiral out of control if we do not develop a new technological ethic. What is right for this kind of responsibility-based environmental ethic that Jonas champions is to promote the interests and needs of future generations. On the other hand, wrong is that which does not promote the conservation of nature and the interests and needs of future generations.

These four schools of thought can very roughly be summarized by four main terms that define them, namely, prudence (*phronesis*), universal rule, consequence, and responsibility. Simply noting the common definition of these four words exemplifies the complex and divergent roads that each ethical analysis would take us to. Should the evolution of biorobots be directed by prudence (avoiding extremes), or should it focus solely on consequences? Should we establish universal rules or laws, or should we prioritize individual or collective responsibility?

To address these queries, we employ a sociotechnical scenario approach, a technique used in science and technology studies to perform ethical analyses of emergent technologies (53, 54). In accordance with the four topics presented above, namely 1) Interactivity, 2) Integrability, and 3) Ontological and moral status of biorobots, we envisage three application areas of biorobots and their ethical challenges. We present three plausible scenarios that align with the potential of this technology and serve as thought experiments. Every individual confronted with these dilemmas might possess a unique viewpoint on how to handle them, likely aligning with one of the four key ethical theories discussed, even if they are not consciously aware of it. While it may be tempting to put on our philosopher’s hat and try to resolve these dilemmas from one perspective or another without reaching a consensus, we prefer to aid researchers in effective reasoning within this space by discussing some of the primary arguments in each scenario and then suggesting a series of CQs they may wish to consider during their research.

#### 2.1.1. Tides and tribulations.

In the near future, a new type of biorobotic device made of invertebrate tissue will be fabricated to clean contaminated waters in the ocean. These robust invertebrate muscles keep the device in motion, while a compartment takes nutrients from the environment to keep them functioning, and another compartment catalyzes chemical reactions that clean microplastics. While scientists ensure that these biohybrid cleaning devices do not travel far from their releasing spot (areas where water is known to be very contaminated) and degrade after some time, they will still enter the food chain of the ocean. Experts warn of the ecological long-term effects of this release, in the form of extinction of some species that ingest them and insertion of microplastics into our food chain.

Many will identify with the utilitarian perspective here as we are presented with a dilemma that might be solved by classical risk analysis. Consequentialism is outcome-centered and holds that positive consequences should be maximized and negative consequences minimized. The question that arises is: What is the lesser evil, the impact of deploying these biocleaners, or the effects of leaving our oceans polluted? On the other hand, deontologism holds that actions that violate a rule or principle are wrong even if they contribute to the greater good. If the risk analysis reflects considerable damage to the environment by the application of these biocleaners (even if it is outweighed by the positive outcomes of cleaning our oceans), the risk taken in the application is simply morally wrong. For some, the acceptable amount of damage to our environment will be exactly zero. Although consequentialism and deontologism often converge and agree, they sometimes diverge, as in this case.

#### 2.1.2. New arms dealing.

In the near future, while the full regrowth of a human arm for those who’ve lost one is still unattainable, strides in human–computer interaction enable the control of artificial limbs using existing nervous tissue. Biohybrid robotic arms, employing actual and adaptable muscles, have progressed to mimic the feel of a real arm. These muscles, however, need to be extracted from living animals, as they are much stronger and more robust than lab-grown tissue. This technological innovation is exceptionally expensive and exclusively accessible to the richest people on Earth. Moreover, these people are using the technology to design their own custom-made limbs, stronger or more flexible than normal arms, to augment human capabilities.

For the ethics of responsibility, technology has acquired a value in itself, as many inventions produce the needs that they satisfy (55). Thus, we must ask ourselves: Is this technology solving a real need that existed before the technology? Certainly, the fabrication of biohybrid arms for people with disabilities would satisfy a real need that existed before the technology. On the other hand, their application for human enhancement would not fulfill any need, and it would be morally wrong to continue since the biosphere (including tissues extracted from animals) is not just an instrument or means for our arbitrary human purposes, but an end in itself. Deontologist views would be concerned about the manner in which the technology is applied and how it impacts its environment. Due to its cost, access to this enhancement technology can only be afforded by a few, giving rise to social divisions and competitive advantages where the people with actual disabilities, originally the intended beneficiaries, might not be able to afford the most emancipating technologies. Therefore, this technology is not fair and, from a deontological point of view, should not be implemented. Even though deontologists and Jonasians may converge here, they might still disagree on developmental decisions within a certain scope (e.g., whether one concern like cost or cell extraction could be overcome).

#### 2.1.3. Machines more like me.

In the near future, biohybrid systems have evolved to create full-sized robots that can interact with humans and perform complex actions in a very organic manner, assisted by muscular tissue and neuromuscular junctions. Every year, they become more complex, and different types of tissues are added into the mix. People buy them and they become helpers around the house and in business. Some people see them as companions, even though they don’t speak. As their complexity increases, people wonder whether they feel pain and are sentient, and how they should interact with them.

Virtue ethics, one of the three major schools of ethical thought (5, 56), places more emphasis on moral character over rules (as deontology does) or act outcomes (as consequentialism does). When faced with the possibility of creating sentient entities (e.g., biorobots), virtue ethics encourages rational action aimed at cultivating the virtues of the entity and ourselves. For instance, avoiding unnecessary harm to biorobots that could be sentient could promote virtues like kindness and compassion and, at the same time, reinforce an ethical practice that, if repeated over time, marks out our character. Our relationship with these new entities should be such that virtues like prudence, justice, righteousness, and compassion are promoted. The Jonasian ethics perspective, as discussed earlier, would not only question the need for this technology but also our moral responsibility toward a potentially new form of life that we have engineered. For Jonas, these biorobots would have an intrinsic value as they are alive, and it is our moral imperative to protect them and not simply use them as means, but as an end in itself.

In conclusion, biohybrid robots, by virtue of their combination of biological and artificial elements, generate unique ethical dilemmas that extend beyond those presented by wholly artificial or biological technologies. The living tissue used in their fabrication, potential for sentience, distinct environmental impact, unusual moral status, and capacity for biological evolution or adaptation are characteristics that distinguish biohybrid robots, inviting novel ethical inquiries. While similarities with other technological ethics can be drawn, these features need a specially tailored ethical framework. Our three scenarios have been designed to capture these unique aspects, presenting situations that encapsulate the distinctive intersection of life and artificiality that is at the core of the technology. As such, they serve as important conversation starters to stimulate discussion on the nuanced ethical landscape of this emerging field. It is imperative for us to proactively confront and navigate these ethical challenges to ensure the responsible and beneficial development of biohybrid robots.

### 2.2. Establishing Actionable Steps.

From these analyses, we can identify certain CQs that seem to emerge from the four philosophical perspectives. People following a utilitarian mindset would be worried about the consequences of these technologies and would make their decision based upon those consequences. Does their impact produce a net positive balance in terms of happiness or pleasure? Further, is the development actually providing pleasure to the biorobots itself, as a potentially sentient being? Deontologists would be worried about universal principles that also consider how the manner in which these technologies are applied impacts their environment. Are these technologies fostering a fairer society or creating more inequalities? Is the activity producing any damage at all to the environment or the biosphere? People aligned with virtue ethics would worry about keeping a prudent balance that would foster values like justice, prudence, or compassion. How is the application of, for instance, biorobotic enhancement making us better human beings? Is it righteous and ethical to create a sentient being for the sake of relieving us from our own work? Am I a responsible scientist if I am not guided by scientific ethics, but by scientific hubris and ambition? Finally, responsible research and innovation makes us think about our real needs and invites us to step back and take a precautionary approach according to practical rationality. Is there a need for the augmentation of human capabilities or the creation of a sentient being, or is it satisfying a need we are creating? Would biorobots have intrinsic value as living beings and should we protect them once they are created?

These critical questions can act as a stimulus for ethical explorations and demonstrate the dynamic and subjective nature of ethics. If we agree on the need to analyze the unique potential dangers of this technology, such as dealing with potentially sentient beings or the usage of living tissues, and establish an international framework for the governance of biohybrid robots, we find that these thought experiments and CQs converge on six necessities of this framework:

1. Perform risk assessments for this technology that consider both the benefits and dangers of its application in different domains.
2. Explicitly consider social implications that are a detriment to democratic values of social justice, fairness, and equality, or that damage our environment and biosphere.
3. Ensure that scientists and innovators appeal to a common good,^#^ not only in relation to humans but also our natural ecosystem, and are adhering to ethical standards accepted in the community.
4. Facilitate interdisciplinary conversations that engage views from (critical) posthumanism or postanthropocentrism to discuss the ontological and ethical status of biohybrid robots.
5. Improve external engagement by inventing new participatory practices to include relevant stakeholders to understand their real needs.
6. Work toward a broader technological literacy among the public to ensure they make informed decisions pertaining to this technology.

These points provide some groundwork for ethical decision-making in biohybrid robotics research. However, ethics is a dynamic field that must evolve with our understanding of technology and its societal impact and, although illustrative, cannot be fully encapsulated by the four schools of thought we have previously discussed. The dynamic nature of ethics, especially in the context of emerging technologies, calls for a more nuanced approach, such as Simone de Beauvoir’s “Ethic’s of ambiguity” (57), which underscores the inherent complexities and uncertainties in ethical decision-making and calls for remaining adaptable and open to diverse ethical viewpoints. For this reason, interdisciplinary conversations should be paramount, in particular with fields such as posthumanism that have been discussing adjacent topics for a few decades (58). Posthumanist views challenge the traditional humanist views of human-technology relationships and could offer fresh insights to navigate our interactions with biohybrid robotics beyond a purely human-centered (anthropocentric) approach. As illustrated by our ample, developments in biohybrid robotics might overlap, unexpectedly, with the inquiries of posthumanist factions like transhumanists, who believe in the inexorable fusion of humans and technology and the augmentation of human capabilities, from artificial limbs to mind uploading into machines (59). Finally, as biohybrid robotics will likely have global implications, international cooperation will be crucial in establishing and implementing these ethical guidelines. The last two of these necessities point toward a final consideration we discuss before concluding: If we carefully consider what is right for biorobotics research ethics and still reasonably disagree, how do we resolve what to do?

## 3. Policy Strategies

The development of biohybrid robots is rapid and we need a collective process that ensures proper use and positive societal effects. Studies of modern governance have developed knowledge of how to effectively and efficiently coordinate a variety of actors with collaborative goals and different individual purposes and responsibilities. This requires joining up thinking from across epistemic communities (e.g., experts), and including all affected in a policy-forming community in an intelligent manner. The challenge is to allow an autonomous, but interdependent, network of researchers and innovators with a plurality of centers of decision-making (e.g., different universities/institutions), to act in an accountable manner (60).

Questions over the rules governing emerging technologies are far from novel. Several cases offer interesting experiences, such as information and communication technology, genetic and reproductive technology, or nuclear energy. No one doubts that emerging technologies must be regulated as they emerge, much like any current technology that can have a societal impact and unintended effects (61). But what form should this regulatory oversight take?

Regulation is a broader concept and is quite controversial in academia (62). Research groups and companies working with biohybrid robotics technology might set rules for themselves, or industry agencies can impose some rules on their members. An open topic of debate is whether self-regulation offers sufficient guarantees against the risks of disruptive and emerging technologies such as biohybrid robotics. And different kinds of regulatory levers exist. Laws, rules, and standards are often used interchangeably when referring to regulation. But there is a spectrum of rules, from laws or legal instruments, to norms: more flexible rules or behaviors often brought about by incentives or sanctioning regimes. Reliance on norms is more necessary in emerging industries where plenty of foresight is needed to understand potential consequences. Whatever levers are used, a reputation for the industry as a responsible and safe sector must be earned.

Some might find calls for greater regulation excessive. We are calling for stakeholder participation in nurturing a culture of philosophical reflexivity, rather than increasing inefficient research bureaucracy that would stifle positive innovation. Scientists in the field should be aware of the risks of not taking the lead in foregrounding ethical regulation. Deference from political actors may seem helpful only until a reactionary moral panic happens. It is better to engender a knowledgeable public as technology develops. If we do not set, maintain, and uphold the highest standards of ethical and responsible research and innovation, we leave ourselves open to catastrophic and unseen failures and moral panics. Without engaging legitimate representatives of wider society to be informed by our efforts, our science is more open to righteous but blunt and overly bureaucratic interventions from external regulatory forces.

The existing research ethics governance regimes are a necessary but not sufficient condition for successful research in this field. In some cases, the field can draw on the extensive work in robot ethics, but the challenge is even more daunting when we talk about biological robots, which are a set of technologies from different disciplines with a range of applications yet to be explored. Because the information asymmetry between those who work on these novel technologies and colleagues even in related fields is so great, democratic governance of biohybrid robots, like other emerging technologies requires new kinds of frameworks, questions, and processes.

Some projects have tried more generally to provide guidance for writing principles for research ethics and activities to be undertaken. The SIENNA project which was completed on March 31, 2021, aimed to analyze ethical and governance issues in three new and developing technological fields: human genomics, human augmentation, and human–machine interaction. In relation to the latter technologies, SIENNA has developed a number of methodologies and tools for discussing the ethical and human rights challenges generated by these technologies, and points particularly to how biorobots are at the forefront of questions about the moral status of robots (63). Moreover, one of the authors of this work has suggested in the past employing current synthetic biology policies and ethical standards as a starting point until biohybrid robots advance further (7).

Any formal governance structure would however only be useful if it can support ongoing negotiation among stakeholders. Deliberative democratic governance would require continuous opportunities for public reasoning, with expectations that outcomes of that process are acted upon (64). Engagement with new participatory democratic practices would allow better anticipation and foresight because it marries disciplinary expertise in biohybrid robotics with other kinds of collective expertise from members of society. Involvement of representatives drawn from the general public in deliberation on ethics would provide knowledge of citizen preferences and information about how potential technological interventions may affect the lives and prospects of ordinary citizens (65). It will also ensure that developments in industry respond to real-world problems, and consider alternative futures. By engaging with wider actors early in the development of emerging technologies like biohybrid robots, not only does development itself benefit from external review, but the technological literacy of the public is improved allowing decisions to be better informed.

The potential misuse of biorobots, especially as dual-use technologies in domains like warfare, underlines the urgent need for robust governance and regulatory frameworks. The challenges in overseeing such technologies are not dissimilar to those encountered in the regulation of autonomous weapons systems and other disruptive technologies like drones. But unlike purely mechanical or digital technologies, biohybrid robots blend biological and synthetic components in unprecedented ways. This unique intersection poses novel challenges in oversight, amplifying the need for dedicated regulatory measures, although we could still draw lessons from these disruptive technologies as a starting point. Thus, the regulation of the creation and application of biorobots might take various forms. To enhance security and ensure ethical application, governments and organizations need to build comprehensive regulatory frameworks that emphasize transparency, proactive regulation, and the importance of ethical standards. In other governance models, regulatory agencies, industry, and researchers would need to collaborate to set standards and best practices for the creation and application of biorobots. For instance, these may include mandatory ethical impact assessments, guidelines for the humane treatment of potentially sentient biorobots, and clear accountability mechanisms for misuse or harm caused by the technology. Now is the optimal time to act on this, and we conclude the discussion by summarizing our contribution and outlining actionable steps researchers can take.

## 4. Conclusion

Development of an emergent technology like biohybrid robotics with potential disruptive applications requires a close inspection of their ethical and social consequences. While this technology has developed relatively unattended by mainstream media, the public, and policy makers, compared to related technologies such as embryonic stem cells or artificial intelligence, it is no less significant. Researchers in the field show welcoming attitudes to socio- and ethico-technical evaluation of this technology, but the first steps in this evaluation process have yet to be started.

The public perception of biohybrid robotics, still unexplored, will be complex and multifaceted, and likely to mirror the discourse around human or animal cloning. There could be potential excitement about its applications in addressing human needs, while on the other hand, significant concerns might arise. One potential response could be what’s been coined the “wisdom of repugnance” or “yuck factor,” which states that an intuitive, deep-rooted negative response to an idea is often evidence of its harmful character. This argument was first articulated by Leon Kass, former chairman of the President’s Council on Bioethics in the United States, to propose a ban on human cloning (66). The consequent debate between bioconservatives, who appeal to our intuitions of repugnance or the “yuck factor,” and bioliberals, who oppose bioconservatives and are characterized as protechnology and in favor of using technology to overcome our limitations, often reaches an impasse, with bioconservatives criticized for cognitive biases and unreliable intuitions (67). While we cannot deny that the concept of biohybrid robotics has been part of our collective imaginations in science fiction (from *Metropolis* to *Blade Runner*), the public’s reaction to the transition of this technology from fiction to reality, especially when considering nonanthropomorphic forms of biohybrid robots, remains to be seen. We call for the general public to be included early in this conversation going forward, ensuring a democratic approach to the development and ethical evaluation of this technology, potentially avoiding entrenched impasses seen elsewhere.

If debates around embryonic stem cells, human cloning, or artificial intelligence have taught us something, it is that humans rarely agree on the correct resolution of the moral dilemmas of emergent technologies. In this work, we have highlighted how biohybrid robotics is not all that different and we can envision different ethical interpretations of feasible scenarios, let alone those currently unimaginable and yet to come. The public, researchers, and policy makers are very capable of intelligent judgment but may lack the vocabulary and lucidity provided by the ethical theories that we have discussed. By close examination and practicing thought experiments, we have identified a set of critical questions that we believe tend to appear in potential applications of this technology and should not be overlooked. Using them as a starting point, we have derived a set of necessities for a framework for research and governance of this technology.

But not all is talk. Actions can be taken at different levels, from research center directors to doctoral candidates, according to their influence:

### 4.1. At the Laboratory Level.

Ensuring students and researchers have Responsible Conduct of Research training. Experiments should be planned with a mindful attitude toward their potential environmental impact and effects on living entities (e.g., animals whose cells are used). It is currently customary in most artificial intelligence conferences to add an ethics statement in papers, disclosing potential (mis)uses of the work that could lead to threatening impact (generally related to data, privacy, and security). Whereas biohybrid robotics pose different risks, we propose the adoption of this custom in papers and conference proceedings, where the authors can discuss ethical considerations of the technology. The project proposal process should also consider these implications and, when possible, aim to collaborate with ethicists and policy experts who can advise the researchers on the implications of their work, giving rise to parallel publications in fields like science and technology studies or public policy.

### 4.2. At the Institutional Level.

Together with the inclusion of ethical statements in research papers, institutions should adapt their institutional review boards to require ethical oversight that recognizes and anticipates real-world and immediate impact of this type of research, which is evolving faster than ever. This should not be translated into an increase of the bureaucratic burden of researchers with new cumbersome processes, but a seamless adaptation of those used in fields like social science, biomedicine, or computer science, where this practice is ubiquitous.

### 4.3. At the Global/National Level.

These efforts would be most useful if internationally coordinated. International conferences could start requiring ethical statements, as machine learning conferences do, to prompt researchers to start thinking about these issues. Specific sessions on ethics and social analysis could be organized, inviting researchers in social science and philosophy to share their views. An international framework for research, governance, and application of biohybrid robots would make sure that the highest standards are being applied and that policymakers are convinced that the dangers are being evaluated while optimizing beneficial impact. This framework would need to allow for the use of modern stakeholder governance, providing democratic oversight to protect and inform researchers and their work, and reducing burdens on individual decision-making.

Taking these steps should not be seen as prescriptive in any way, but as an opportunity to share responsibility, taking a heavy weight away from the researcher’s shoulders. As researchers, we are members of a community, that also includes social scientists and ethicists, involved in the sociotechnical analysis of this technology. It would be an opportunity for collaborations and interdisciplinarity to flourish, improving the engagement with stakeholders, ranging from the general public to policymakers. Research in biohybrid robotics has evolved in various directions for some time, and we need to align our efforts to fully unlock its potential.

## Data Availability

There are no data underlying this work.

## Footnotes

^*^We used the following keywords for our search: “biohybrid system$” OR “bio-hybrid actuator$” OR “biohybrid actuator$” OR “cell-based actuator$” OR “bacteria robot$” OR “bacteriabot$” OR “bacteria-bot$” OR “muscle bioactuator$” OR “muscle-based bioactuator$” OR “muscle-based bio-actuator$” OR “bioactuator$” OR “bioactuator$” OR “biohybrid robot$” OR “bio-hybrid robot*” OR “hybrid bio-robot*” OR “hybrid biorobot*” OR “bio bot$” OR “bio-bot$” OR “biobot$” OR “xeno-bot$” OR “xenobot$” OR “bio-hybrid device$” OR “biohybrid device$” OR “bio-hybrid system$” OR “biohybrid system$.” Here, the $ sign means “any zero or one character” to account for plurals, and the * sign means “any combination of characters” to account for constructions like “robotic.” We purposely avoided terms like “cyborg” or “synthetic biology” since, although they are related, they are generally not the primary terms used by researchers in biohybrid robotics, as they’re generally reserved for fields like transhumanism or genetic engineering and not robotics.

^†^The other ones were related to neuroprostheses, artificial blood feeders for mosquitos, 3D organoids, dentistry, AI, and another one was a completely inaccessible proceedings paper.

^‡^With keywords like “social” and “implication*,” there were several false positives, for instance, papers discussing “social robots” or the implications of clinical research using bacteria. We are only focusing on the ethical implications and, for the sake of space, are not delving deeper to discuss the other keywords. However, even considering false positives, we demonstrate a rather low number of papers discussing the sociotechnical evaluation and implications of this technology.

^§^Researchers studying consciousness have not yet agreed on a theory and explanation of consciousness, but a very simple and comprehensive definition of consciousness is any subjective experience, whether it is a visual experience of the surrounding world, experience of emotions, etc. (52).

^¶^Normative ethics is a branch of moral philosophy that aims to establish principles or criteria guiding our actions.

^#^The concept of “common good” in political philosophy refers to what communities do together for the benefit of everyone. Canonical examples of this include infrastructures and services like roads, parks, public safety, clean water… This idea highlights that citizens are in a civic relationship that requires them to make decisions that are for the benefit of everyone, not only a few individuals. Here, we also expand the “common good” to cover the natural environment, including the animals that are used to extract cell lines and, potentially, the biohybrid robots themselves.
